# Microdebrider-Assisted Rhinophyma Excision

**DOI:** 10.1155/2019/4915416

**Published:** 2019-11-20

**Authors:** Abdulaziz Abushaala, Marios Stavrakas, Hisham Khalil

**Affiliations:** ENT Department, University Hospitals Plymouth NHS Trust, Plymouth, UK

## Abstract

Rhinophyma represents a progressive deformity of the nose which leads to cosmetic disfigurement and has a significant impact on the patient's quality of life. This pathological entity originates from hyperplasia of sebaceous gland tissue, connective tissue, and vessels of the nose and is associated with rosacea and more specifically, stage III rosacea. Surgical treatment is the method of choice. We present five cases of rhinophyma that we treated with microdebrider-assisted excision. The procedure was divided in two main steps: scalpel excision of the main bulk of the rhinophyma and then further contouring with the microdebrider. All patients had weekly follow-up for the first four weeks, and then three-monthly. All patients had uneventful recovery and satisfactory cosmetic outcomes. No postoperative infections or other complications were reported in our case series. The use of the microdebrider reduces the operating time, preserves the islands of skin regeneration, and allows finer manipulations than the standard scalpel techniques. Microdebrider-assisted rhinophyma excision is a safe approach, with good aesthetic results. Larger series of patients need to be examined in order to establish the value of the method.

## 1. Introduction

Rhinophyma is a rare condition, affecting predominantly Caucasian males, with a male-to-female ratio of 12–30 : 1 [1–4]. Despite the fact that the pathology is known in the medical world since 1845 (term introduced by Hebra, deriving from the Greek synthetics “rhis” and “phyma”, which means growth of the nose [1, 5]), its exact pathogenesis still remains unclear De novo rhinophyma is also well recognized. It tends to occur in age above 50 years and is a benign dermatological disease, with cosmetic, functional, and psychosocial effects. Various aetiopathogenic theories have been presented and even more treatment modalities have been reported, from medical therapy for early stage disease to different surgical excision techniques.

We present our experience of five rhinophyma cases treated in our department using the microdebrider-assisted technique, comprising a combination of partial thickness skin excision with a scalpel followed by microderider final contouring.

## 2. Materials and Methods

Five patients were diagnosed with rhinophyma between the years 2010 and 2018. They were all White British males between 64–77 years of age. The main complaint of all patients was cosmetic unsightly appearance of the nose; however, four patients complained also of nasal airway obstruction. One patient admitted the psychological impact of the deformity. Two of the patients in the study group underwent revision surgery, having had primary surgery at a different institution, one with laser and the other with simple scalpel excision. The duration of symptoms varied between 2 and 6 years, with an average of 4 years. None of the patients had history of rosacea. One patient admitted ex-heavy alcohol consumption, one moderate drinking, and three nondrinkers. The deformities have mainly affected the dorsum of the nose; however, it also involved the tip, dorsum, and left alar subunit in one of the recurrence cases (Table 1).

All five patients underwent microdebrider-assisted rhinophyma excision as day-case surgery. After the initial consultation and surgical planning, they were seen once again at the consent clinic prior to the day of surgery and preoperative clinical photographs (standard nose series) were obtained. Under general anaesthesia, the initial excision of the main rhinophyma bulk was performed using a no. 10 blade, aiming to respect the nasal subunits. This first stage was followed by microdebrider contouring, using a straight microdebrider handpiece (Medtronic Straightshot M4 Microdebrider). The surgeon paid attention to avoid extensive damage to the deep dermis that will eventually enable skin regeneration. Also, great care was taken not to destruct the underlying cartilaginous structures. Haemostasis was achieved with bipolar diathermy, direct pressure, and topical application of adrenaline 1 : 10.000. Excised tissues were subjected to histopathological analysis. The raw area was covered with Jelonet paraffin gauze and chloramphenicol ointment, and the patients were discharged on a week's course of broad-spectrum oral antibiotics. All patients had weekly follow-up for the first month and then three-monthly follow-up appointments up to 12 months, when they were discharged.

## 3. Results

**Figure 1:**
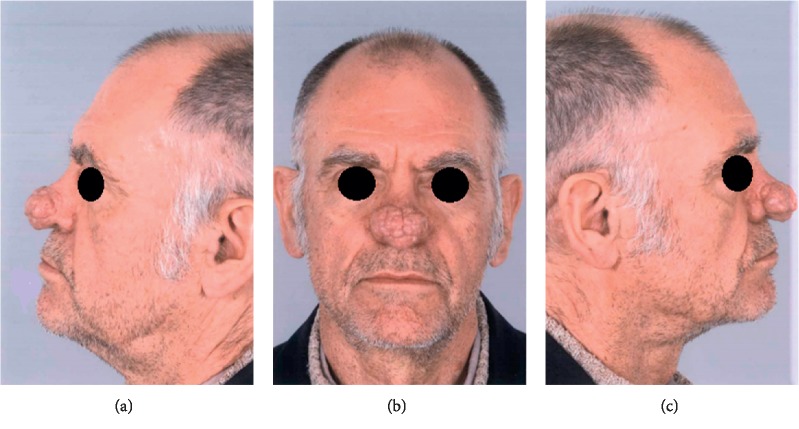
Preoperative presentation. Obvious deformity of the nasal tip and dorsum, also causing nasal obstruction.

**Figure 2:**
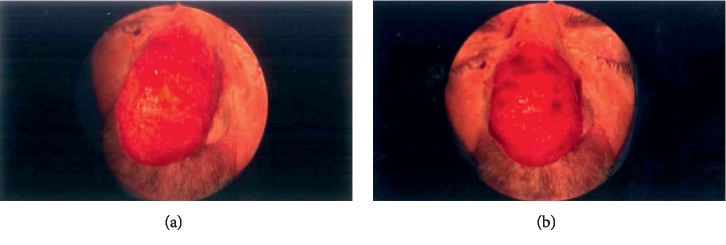
Intraoperative photographs after the final sculpturing using the macrodebrider.

**Figure 3:**
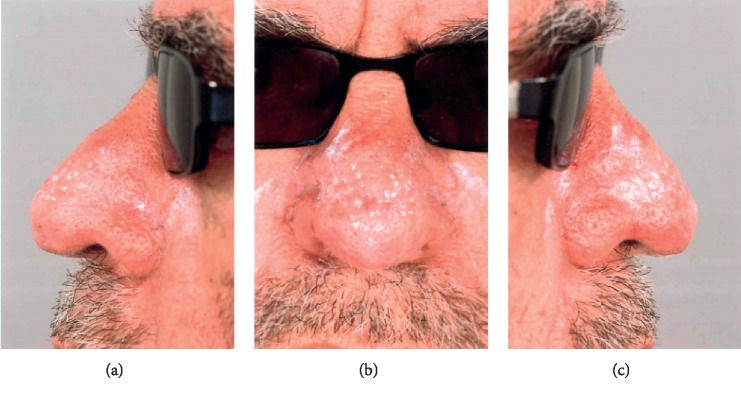
Postopeartive photographs, showing the final outcome after the initial healing period.

The most common histopathological findings included nodular pieces of skin with dermal fibrosis and dilated, plugged hair follicles together with sebaceous gland hyperplasia. No dysplasias or malignancies were identified in our case series. All patients were pleased with their outcomes, they were more socially active, and their confidence improved. No recurrence was reported in our study group, and the patients did not require any additional medical treatment or any further aesthetic surgical procedures. (Figures 1–3)

## 4. Discussion

Rhinophyma is closely associated with acne rosacea and more specifically, type III of acne rosacea [6, 7]. Another possible aetiologic factor is colonization of the sebaceous glands with *Demodex folliculorum* [6, 8]. None of the above has been proven to be an established causative factor for the formation of rhinophyma. Moreover, there is a discrepancy between the age and gender affected by rosacea and rhinophyma. The former affects mainly women of a younger age, while the latter affects elderly male patients [6].

The surgeon should keep in mind the incidence of basal cell carcinoma (BCC), which has been reported to be between 5% and 10% in the rhinophymatous tissue [1, 9]. Other pathologies in the differential diagnosis are squamous cell carcinoma (SCC), sebaceous carcinoma, angiosarcoma, metastatic disease, granuloma eosinophiliacum, sarcoidosis, and lymphoma [4, 10–16].

Medical treatment has been employed, especially in the early stages of rhinophyma, aiming to slow down its progression. Low-dose isotretinoin has been used in the management of early rhinophyma [17]. Another potentially useful strategy is the administration of tamoxifen, as it is proven to reduce the production and secretion of TGFb2 by rhinophyma-associated fibroblasts [18, 19]. There is no solid evidence in the literature confirming that the abovementioned treatments are successful.

The mainstay in the treatment of rhinophyma is surgery. Today, there are numerous methods available, but unfortunately, the literature is lacking large series and safe conclusions on the outcomes of each method. The principles of surgical treatment are similar, based on the excision of the nasal skin and secondary healing of the defect.

Since the first treatment of rhinophyma by Dieffenbach in 1845 (excision and primary closure) and von Langenbeck in 1851 (excision and secondary healing), several methods have been introduced, and the basic axons of development are haemostasis and bloodless surgical field, satisfactory aesthetic outcome, and reduction of surgical time [4]. Cryosurgery was first introduced in 1970 as a treatment option for rhinophyma [20]. Main advantages are minimal bleeding, little pain, and no destruction of the underlying nasal cartilages if used appropriately. On the other hand, it can result is dyschromia and scarring [21]. Blade excision remains one of the widely used methods. A modification is the Shaw heated scalpel which cuts tissue and coagulates blood vessels at the same time. Disadvantages of this method are postoperative pain, mild scarring, and slight nasal alar collapse [22]. Another heat-delivery instrument that has been successfully used is Coblation. It raises the temperature to 60°C–70°C, compared to the Shaw blade, which reaches temperatures from 150°C to 200°C. In that way, Coblation ensures bloodless field and minimal pain. Disadvantages of the method are hypopigmentation and prolonged erythema [23, 24]. Other surgical methods include the use of the harmonic ultrasound scalpel [25], dermabrasion [26], electrosurgery [27, 28], and Versajet hydrosurgery system [29]. In addition, laser-assisted treatments have been described, including Co2 laser [30–32], Erbium:YAG laser [33, 34], and the diode laser [35, 36]. Also, grafting techniques and the subunit method have been used. The latter aims to address the hypertrophic sebaceous tissues, the excess skin problem, and also the destruction of support [37].

Microdebrider-assisted rhinophyma excision is not a new method in the literature, although few cases have been presented up to date. Farris et al. presented three cases treated with blade excision and microdebrider sculpturing or microdebrider excision alone [38]. We have followed a two-stage approach in the five cases that we present. Scalpel blade excision allows quick removal of the main tissue bulk and obtaining of tissue samples for histology, while the use of the microdebrider is beneficial for quick contouring and shaping of the nasal subunits, reducing the risk of damage to the underlying cartilaginous structures. We believe that a straight microdebrider handpiece is appropriate for tip refinement and work around the nasal alae. The preservation of deeper skin layers allows re-epithelialisation with less scarring and also the tactile feedback that the surgeon takes from the instrument. As it is a relatively quick method, it reduces the intraoperative blood loss and the risk of infections. All our patients recovered well, with no postoperative complications and were satisfied with the aesthetic outcome. Our case series needed revision surgery, either for recurrence or aesthetic improvements.

We should not forget the psychological impact of rhinophyma and the stigmatization it may bring about to the patients. A study by Bohm et al. revealed higher risk of depression and anxiety among patients suffering from rosacea or rhinophyma compared with the general population. Especially patients with rhinophyma had more chances to be rejected by others and felt disfigured. Obviously, all the abovementioned lead to the conclusion that acne rosacea and rhinophyma can affect the patients' quality of life [39]. The clinician should always keep that in mind and consider psychiatric cotreatment if necessary. All our patients felt significant psychological improvement after the recovery period.

Finally, an equally important issue when it comes to method selection is cost of the equipment used. Nowadays, costs represent a main concern in many countries, and there is a variable set of circumstances in which potentially scarce equipment often force surgeons to choose only the best option available. We believe that microdebrider-assisted rhinophyma excision is cost effective, considering that it requires less theatre time and the equipment used is nonspecific and readily available in most places with a general rhinology service [40].

## 5. Conclusion

Rhinophyma has a quite rare pathology with functional, aesthetic, and psychological impact. The problem should be evaluated in its entirety and addressed in the most appropriate way according to the surgeon's opinion. A number of techniques are available including the microdebrider-assisted excision. This method is relatively fast, allows fine sculpturing, and leaves adequate tissue sample for histology. The postoperative results are encouraging, there is a low recurrence rate, and the cosmetic result is satisfactory. We also believe that this technique can be easily mastered by surgeons who have experience in the use of microdebriders.

## Figures and Tables

**Table 1:** Patient characteristics and symptoms on presentation.

	Sex	Age	Duration of symptoms (years)	New/recurrence	Nasal obstruction	Main symptom	Deformed nasal subunits
1	M	77	3	New	Yes	Nasal deformity, obstruction, and psychological effect	Tip
2	M	64	2	New	No	Nasal deformity	Tip
3	M	66	5	Recurrence	Yes	Nasal deformity and obstruction	Tip, dorsum, and left alar subunit
4	M	71	6	Recurrence	Yes	Nasal deformity and obstruction	Tip
5	M	70	4	New	Yes	Nasal deformity and obstruction	Tip
